# Regeneration of hyaline-like cartilage in situ with SOX9 stimulation of bone marrow-derived mesenchymal stem cells

**DOI:** 10.1371/journal.pone.0180138

**Published:** 2017-06-30

**Authors:** Xiaowei Zhang, Shili Wu, Ty Naccarato, Manan Prakash-Damani, Yuan Chou, Cong-Qiu Chu, Yong Zhu

**Affiliations:** 1 Division of Arthritis and Rheumatic Disease, Oregon Health & Science University, Portland, OR, United States of America; 2 Section of Rheumatology, VA Portland Health Care System, Portland, OR, United States of America; 3 VivoScript, Inc, Costa Mesa, CA, United States of America; University of Connecticut Health Center, UNITED STATES

## Abstract

Microfracture, a common procedure for treatment of cartilage injury, induces fibrocartilage repair by recruiting bone marrow derived mesenchymal stem cells (MSC) to the site of cartilage injury. However, fibrocartilage is inferior biomechanically to hyaline cartilage. SRY-type high-mobility group box-9 (SOX9) is a master regulator of chondrogenesis by promoting proliferation and differentiation of MSC into chondrocytes. In this study we aimed to test the therapeutic potential of cell penetrating recombinant SOX9 protein in regeneration of hyaline cartilage *in situ* at the site of cartilage injury. We generated a recombinant SOX9 protein which was fused with super positively charged green fluorescence protein (GFP) (scSOX9) to facilitate cell penetration. scSOX9 was able to induce chondrogenesis of bone marrow derived MSC *in vitro*. In a rabbit cartilage injury model, scSOX9 in combination with microfracture significantly improved quality of repaired cartilage as shown by macroscopic appearance. Histological analysis revealed that the reparative tissue induced by microfracture with scSOX9 had features of hyaline cartilage; and collagen type II to type I ratio was similar to that in normal cartilage. This short term *in vivo* study demonstrated that when administered at the site of microfracture, scSOX9 was able to induce reparative tissue with features of hyaline cartilage.

## Introduction

Articular cartilage defect is extremely common and carries a remarkably higher risk to progress to osteoarthritis (OA) [1–3]. Repair of defective articular cartilage remains a challenge. Mature joint cartilage is free of blood vessels and enervation. This makes articular cartilage a tissue with an almost absent intrinsic repair capacity in most *in vivo* situations. The commonly practiced surgical procedure, microfracture induces migration of bone marrow mesenchymal stem cells (MSC) to the site of cartilage defect. By drilling small holes deep into the subchondral bone marrow space, microfracture induces bleeding of bone marrow and form clot at the surface of cartilage defect. It is thought that the MSC contained in the bone marrow clot proliferate and differentiate into chondrocytes [4]. Clinical studies indicate that microfracture provides effective short-term improvement of joint function but with shortcomings of poor long-term improvement and possible functional deterioration after 24 months [4]. This is mainly because of fibrocartilage or fibrohyaline hybrid tissue rather than hyaline cartilage was generated by this procedure. Fibrocartilage contains less proteoglycan and more type I collagen with inferior biomechanical property [4, 5]. Therefore, it is an unmet need for development of a method to induce hyaline cartilage *in situ*.

SOX9 is a member of the family of SOX (SRY-type high-mobility group (HMG) box) genes and has been shown to be expressed predominantly in mesenchymal condensations and cartilage [6, 7]. SOX9 acts with different partners to orchestrate activation and repression of transcription in the chondrogenic differentiation pathway. Thus SOX9 plays an essential role as a transactivator of many genes preferentially expressed in non-hypertrophic chondrocytes such as *Col2a1* and aggrecan gene promoter/enhancer [8, 9]. Concomitantly, SOX9 directly represses expression of *Col10a1* at a stage prior to the onset of hypertrophy [10]. In humans, haploinsufficiency of SOX9 results in campomelic dysplasia [6]. In mice, no cartilage developed in tetratomas derived from SOX9 knocked out embryonic stem cells [6]. Transgenic over-expression of SOX9 in hypertrophic chondrocytes causes down-regulation of collagen type X [10]. All these data indicate that SOX9 is a master regulator of the chondrocyte phenotype. Most importantly, while promoting chondrogenesis, SOX9 can concomitantly repress chondrocyte hypertrophy. In addition, SOX9 also inhibits osteogenesis by suppressing collagen type I and osteocalcin [11, 12]. SOX5 and SOX6 co-operate with SOX9 in promoting chondrogenesis of MSC. Since MSC normally express both SOX5 and SOX6 and SOX9 further upregulates SOX5 and SOX6 expression [13], promotion of SOX9 expression alone is sufficient to direct MSC differentiation into chondrocytes. This has been confirmed by *in vitro* cultures where lipofection mediated SOX9 gene transfection alone in bone marrow derived MSC successfully differentiate into chondrocytes with no additional growth factors added [14]. Moreover, adenovirus mediated SOX9 gene transfer enables articular cartilage regeneration *in situ* in a rabbit model of cartilage defect repair [11], but viral vectors suffer from inherited disadvantage of being potentially causing infection by the vector virus and hence it is not suitable for clinical use [11].

To overcome problems with gene therapy involving viral vectors, we took a novel strategy to provide SOX9 protein to MSC for promotion of chondrogenesis *in vivo* with a simple one stage method. Proteins are large molecules which are unable to enter cells spontaneously. This has limited the use of intracellular proteins as therapeutics. However, recent advancement in protein engineering has shown that functional proteins can be delivered into mammalian cells *in vitro* and *in vivo* using supercharged proteins [15–19]. We have produced a bioactive supercharged SOX9 (scSOX9) which has the capability of entering cells and inducing MSC to differentiate into chondrocytes *in vitro*. Here we report that it was technically feasible to administer this scSOX9 at the site of microfracture and scSOX9 significantly improved quality of repaired hyaline cartilage.

## Materials and methods

All methods were performed in accordance with the approved guidelines. All animal studies were approved by Oregon Health & Science University Institutional Animal Care and Use Committee (protocol number: IP00000169).

### Reagents

All the media, buffers, supplements, and reagents for cell culture were obtained from GIBCO BRL-Life Technologies (Grand Island, NY) or Sigma Chemical Co. (St. Louis, MO). Super positively charged green fluorescence protein (scGFP) and scSOX9, scSOX9-A76E and scMyoD were kindly provided by VivoScript, Inc. (Costa Mesa, CA).

### Gene construction and protein production of recombinant scSOX9

A novel approach was designed to produce a bioactive SOX9 protein with the property of cell penetration. This was achieved by molecular engineering, i.e., fusion of SOX9 with scGFP and addition of a polyarginine tag (11R) [20]. The fusion protein is referred as super-charged SOX9 (scSOX9). When GFP was modified to be super positively charged, it can penetrate mammalian cells. The internalization depends on nonspecific electrostatic interactions with sulfated proteoglycans present on the surface of most mammalian cells [17]. When fused to super positively charged GFP, proteins rapidly enter mammalian cells with potency up to 100 folds greater than that of corresponding fusions with known protein transduction domains including Tat, oligoarginine and penetratin [16]. The super positively charged GFP moiety of the fusion protein serves two functions: transmembrane cell penetration and direct visualization of the fusion protein intracellularly.

### Cell culture and penetration of scSOX9 protein

Human skin fibroblast cell line, HFF or human bone marrow derived MSC at passage 5 were grown in monolayer in Dulbecco’s Modified Eagle’s Medium (DMEM) containing 10% FBS and low glucose (1.5 g/l). The cells cultured in 96 well flat-bottomed plates were incubated with 10 μg/ml of scGFP or scSOX9 at 37°C for 1 hour. Cells were washed with PBS containing 20U/ml heparin (pH7.4) to remove cell membrane bound scSOX9 and directly viewed under fluorescent microscope to visualize transmembrane cell penetration.

Human MSC treated with or without 10 μg/ml of scSOX9 were washed, trypsinized and analyzed in flow cytometry to further confirm scSOX9 cell penetration by mean fluorescence intensity (MFI). MSC treated with or without scSOX9 were washed with PBS containing heparin and permeabilized, followed by incubation with streptavidin-Cy3 (red) (streptavidin binds to positively charged) and viewed under a confocal microscope. The nucleus was stained blue with DAPI.

### scSOX9 induced proliferation and chondrogenesis of MSC

MSC were cultured in DMEM containing 10% FBS and low glucose (1.5 g/l) only or with addition of scGFP or scSOX9 or a cocktail of growth factors (GF) (containing 10% of ITS Premix Tissue Culture Supplement (Becton Dickson), 10^−7^ M dexamethasone and 10 ng/ml of transforming growth factor-ß1) for 48 h [21]. The proliferation of MSC was evaluated by cell counting. Human bone marrow MSC were cultured in DMEM Medium with high glucose (4.5g/l) (DMEM-HG) in a 96 well flat-bottomed plate. The cells were treated with or without 10 μg/ml scSOX9 or a cocktail of growth factors in high glucose DMEM containing 1% FBS. After 24 hours, culture medium was changed to DMEM containing 10% FBS and high glucose with no additional scSOX9 was added. Culture was maintained for 21 days. MSC were stained with Toluidine blue on plate for aggrecan at 7 day, 14 days, and 21 days.

### scSOX9 delivery into MSC by a bilayer collagen membrane

We next tested the bioactivity of scSOX9 *in vivo* in a rabbit cartilage injury model. A commercial bilayer collagen membrane (Bio-Gide, Geistlich Pharma AG, Switzerland) was proposed to serve as a carrier for scSox9 to be administered at the site of microfracture. We first tested the collagen membrane for adhesion and release of scSox9. A collagen membrane at 4 mm in diameter was soaked in 25 μl of 100 μg/ml of scSOX9 solution for one hour. Green fluorescence was grossly visible on the collagen membrane. The membrane was taken out and scSOX9 protein remained in the solution was measured with a spectrophotometer at 497 nm; scSOX9 bound to the membrane was calculated. Collagen membrane bound scSOX9 was rinsed with PBS containing 20 units/ml of heparin (pH7.4) for 1 hour. The amount of scSOX9 released from the membrane was calculated by measuring scSOX9 in the solution.

The efficiency of scSOX9 delivery into MSC *in vivo* was assessed. A cylindrical osteochondral defect (also referred as cartilage defect in the literature) of 4 mm in diameter and 3 mm in depth was created in patellar groove of the femur as described [11, 22, 23]. Microfracture was created using 0.9 mm Kirschner wire (see details below) and bleeding of bone marrow was allowed to fully fill the defect. A collagen membrane harboring scSOX9 was secured to cover the defect. One hour later, the bone marrow clot from the defect was harvested, minced and digested with streptokinase as described [24]. An average, 30 μl of bone marrow clot was recovered from each defect (the calculated volume of each defect was 37.68 μl). The digested bone marrow cell suspension was washed with PBS containing 20 units/ml of heparin to eliminate cell membrane bound scSOX9, and stained with antibodies against CD90-APC, CD11b-PE, CD79a-PE and MHC-DR-PE (Ad Serotec) for 30 minutes. After red blood cells were lysed the cells were analyzed on flow cytometry for delivery of scSOX9 into MSC. MSC was defined as CD90^+^/CD11b^-^/CD79a^-^/DR^-^. GFP positive cells indicated that scSOX9 entered MSC.

### Real time RT-PCR and immunohistochemistry

Human bone marrow MSC were cultured with DMEM containing 1% FBS and high glucose (4.5g/l) with addition of 10 μg/ml of scGFP or 10 μg/ml of scSOX9 or a cocktail of growth factors (GF) for indicated time points. RNA was extracted and RT-PCR was performed with TaqMan probe based analysis assay for collagen (Col) type I, II and X mRNA expression. Briefly, total RNA is extracted and contaminating DNA is removed. A two-step RT-PCR reaction employed 200 ng total RNA per 10 μl reaction with a mix of random and oligo-dT primers, using SuperScript III Reverse Transcriptase. RT-PCR is performed and primers for gene expression is purchased pre-designed from Life Technology (collagen type I COL1A1 Hs00164004_m1, COL2A1 Hs00264051_m1, COL10A1 Hs00166657_m1, SOX9 Hs01001343_g1, SOX5 Hs01425825_cn and SOX6 Hs00264525_m1). The efficiency of amplification is determined for each primer set from serial dilutions. mRNA expression was relative to GAPDH; scSOX9 and GF treated were compared to scGFP treated. Data are presented as fold change relative to control samples. mRNA expression is normalized to GAPDH [25]. At day 14, aggregates were harvested and snap-frozen. Matrix collagen content expression at protein level in cells was analyzed by immunohistochemistry. Cryostat sections were blocked with PBS containing 2% normal goat serum for 1 hour, then incubated with a mouse anti-human collage II monoclonal antibody for 16 hours at 4°C. This was followed by a secondary antibody conjugated with horseradish peroxidase. Staining was developed with peroxidase substrate AEC.

### Osteochondral defect creation and microfracture

Rabbit model is commonly used in experimental cartilage injury and repair studies [11, 22, 23, 25, 26]. Mature female New Zealand white rabbits with body weight of 3.5–4.0 kg are used in the study. Under intravenous anesthesia with 25 mg/kg pentobarbital, a medial para-patellar skin incision is made to the right knee, and the knee joint is exposed via lateral dislocation of the patella. A cylindrical osteochondral defect of 4 mm in diameter and 3 mm in depth is made at the patella groove of femur using a dermal biopsy stainless steel punch and manual debridement as described [25, 26]. Microfracture is performed using a 0.9 mm Kirschner wire tapped into the subchondral bone to a depth of approximately 3 mm until bleeding from the hole is apparent [25, 26]. Three microfracture holes are created within each full thickness chondral defect in a triangular configuration. The defect of cartilage was either left with no treatment, treated with microfracture or microfracture with scSOX9 bound collagen membrane (S2 Fig). A supercharged recombinant scSOX9 mutant, scSOX9-A76E, or scMyoD (myogenic differentiation factor) bound collagen membrane, or collagen membrane only was used as controls. Rabbits were set for free in movement and observed for 8 weeks without restriction of movement.

### Gross morphology assessment

Rabbits were euthanized by intravenous overdose of pentobarbital 8 weeks after surgery. The entire knee was dissected and distal part of the femur was then extirpated. The samples from each group were examined and photographed for evaluation in a blinded manner according to the International Cartilage Repair Society (ICRS) macroscopic assessment scale for cartilage repair (S1 Table) [27]. Percentage of the defect surface area covered with repaired tissue is calculated as follows:

(originaldefectarea)–(defectareaatsacrifice)/(originaldefectarea)*100

### Histological assessment

After gross examination, specimens were fixed in 10% buffered formalin for 48 hours, decalcified in Calci-Clear (National Diagnostics) and then embedded in paraffin. Serial sections of 5 μm were cut horizontally along the maximum diameter of the repaired sites. Sections were stained with hematoxylin and eosin and Safranin O and fast green using standard protocols. Histological analysis of cartilage repair was scored as described. Each of the categories was reported separately according to the ICRS recommendation (S2 Table) [28, 29]. Safranin O staining was assessed as described [30]. Histological analysis and safranin O staining were scored in a blinded manner. The stained sections were coded for their *in vivo* treatment by an independent laboratory technician (LL) and the coded sections were scored by two investigators (XZ and CQC).

### Quantification of collagen contents in reparative tissue

To quantify the collagen contents, full thickness of cartilage was harvested using a curette from each repaired defect of rabbit distal femurs. About 2 mg of cartilage was collected from each area of cartilage repair. The cartilage was snap frozen in liquid nitrogen and ground. This was followed by treatment with guanidine hydrochloride to remove proteoglycan, then by digestion with pepsin and elastase to solubilize collagen. Collagen type II and type I in the reparative tissue were determined by ELISA Collagen Detection kits (Chondrex, Redmond, WA), according to manufacturer instructions and as described [31]. All incubation procedures were performed at 4°C.

### Statistics

Gross and histological scores were graded in a blinded manner and analyzed with one-way ANOVA followed by post hoc analysis with Newman–Keuls Multiple Comparison Test. Student t test was applied for analysis of real-time PCR data. Differences of p<0.05 were considered statistically significant. Analyses for gross morphology, histological assessment including matrix content, and collagen type for the regenerated tissue at the sites of cartilage defect were directly compared with normal articular cartilage adjacent to the defect sites.

## Results and discussion

### Penetration of scSOX9 Protein into HFF and MSC and promoted gene expression

A bioactive SOX9 protein with the property of cell penetration was achieved by molecular engineering. As shown in Fig A-D in S1 Fig, scSOX9 readily entered human foreskin fibroblast (HFF) cells and bone marrow derived MSC. After one hour incubation with scSOX9, both HFF cells and MSC showed intracellular expression of green fluorescence indicating entry of scSOX9 into these cells. scSOX9 sustained in intracellular compartment of MSC for up to 7 days (Fig E and F in S1 Fig). Most of the full-length proteins are trapped in endosomes for days. Some are cleaved and the SOX9 moiety escaped from endosomes and translocated into the nuclei [16]. The green fluorescence may indicate the pool of full-length scSOX9 in the cytoplasm. On average scSOX9 was readily delivered into 96 ± 3% of MSC after 1 hour incubation (Fig G in S1 Fig). Upon internalization, the GFP moiety was cleaved off and retained in endosome. The 11 arginine (11R) facilitates scSOX9 transduction into subcellular compartments and nucleus. As shown in Fig H in S1 Fig, scSOX9 entered nucleus of MSC after GFP was off. Functional property of scSOX9 has been further verified by *in vitro* cell culture of human bone marrow derived MSC for their differentiation into chondrocytes. Possibly owing to its two intrinsic nuclear-localization signals (NLS) and 11R tag, scSXO9 readily enters nucleus.

### scSOX9 upregulated proliferation and chondrogenesis of MSC

**Fig 1:**
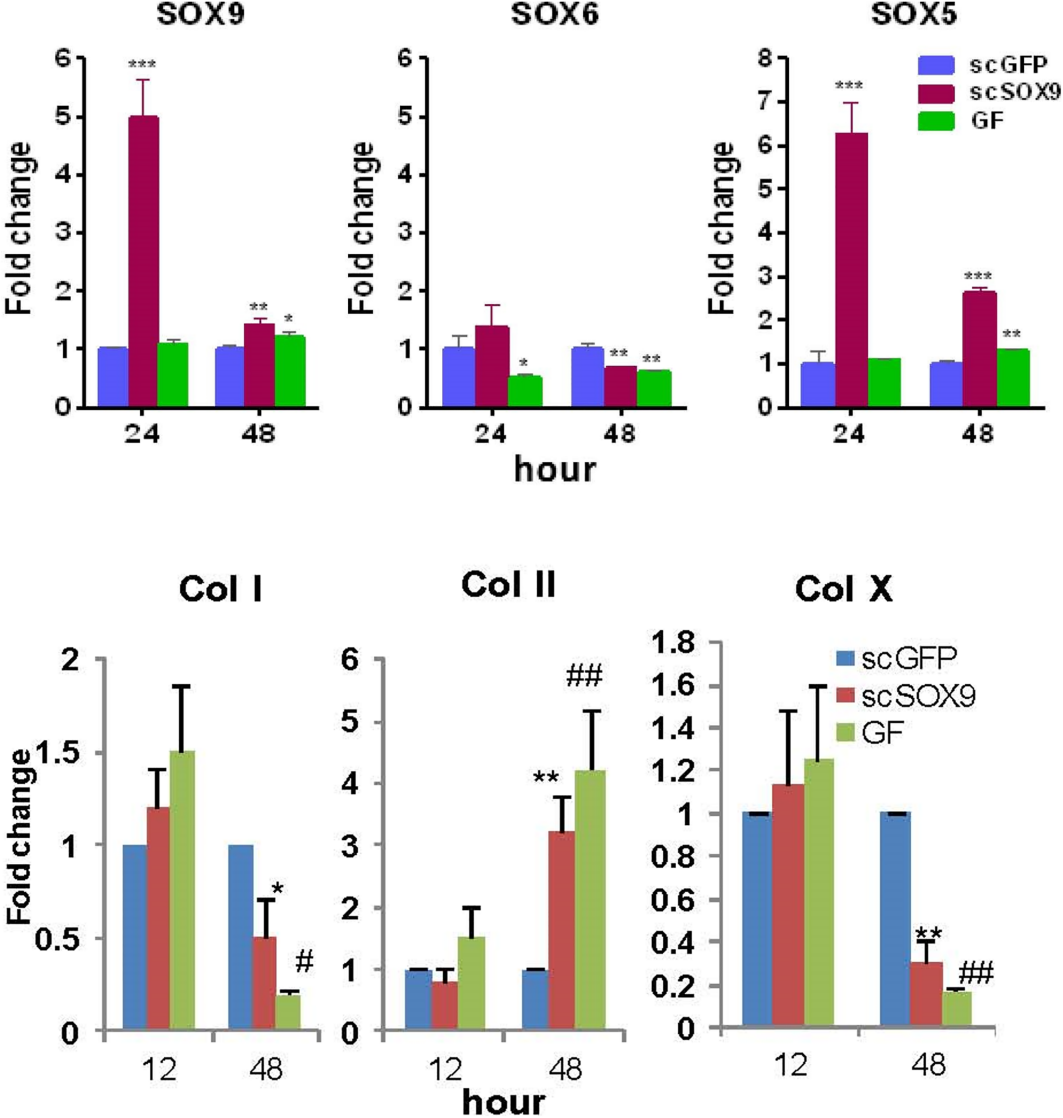
scSOX9 induced increased expression of SOX5, SOX9 and collagen II but decreased expression of collagen I and X. Human bone marrow derived mesenchymal stem cells (MSC) were cultured with DMEM containing 1% fetal bovine serum and high glucose (4.5g/l) with addition of 10 μg/ml of scGFP or 10 μg/ml of scSOX9 or a cocktail of growth factors (GF) (see Material and Methods). RNA was extracted and RT-PCR was performed with TaqMan probe based analysis assay for mRNA expression of SOX5, SOX6 and SOX9 (A); and collagen (Col) type I, II and X (B). mRNA expression of collagen type I, II, X, SOX5, SOX6 and SOX9 was relative to GAPDH; scSOX9 and GF treated were compared to scGFP treated. 1A: * p <0.05; ** p < 0.01; *** p < 0.001. 1B: *p = 0.04, ** p = 0.001, #p = 0.02, ##p = 0.006 (n = 4 in each time points). SOX: SRY-type high-mobility group box; scSOX9: super-charged SRY-type high-mobility group box-9; scGFP: super-charged green fluorescence protein; Col: collagen.

**Fig 2:**
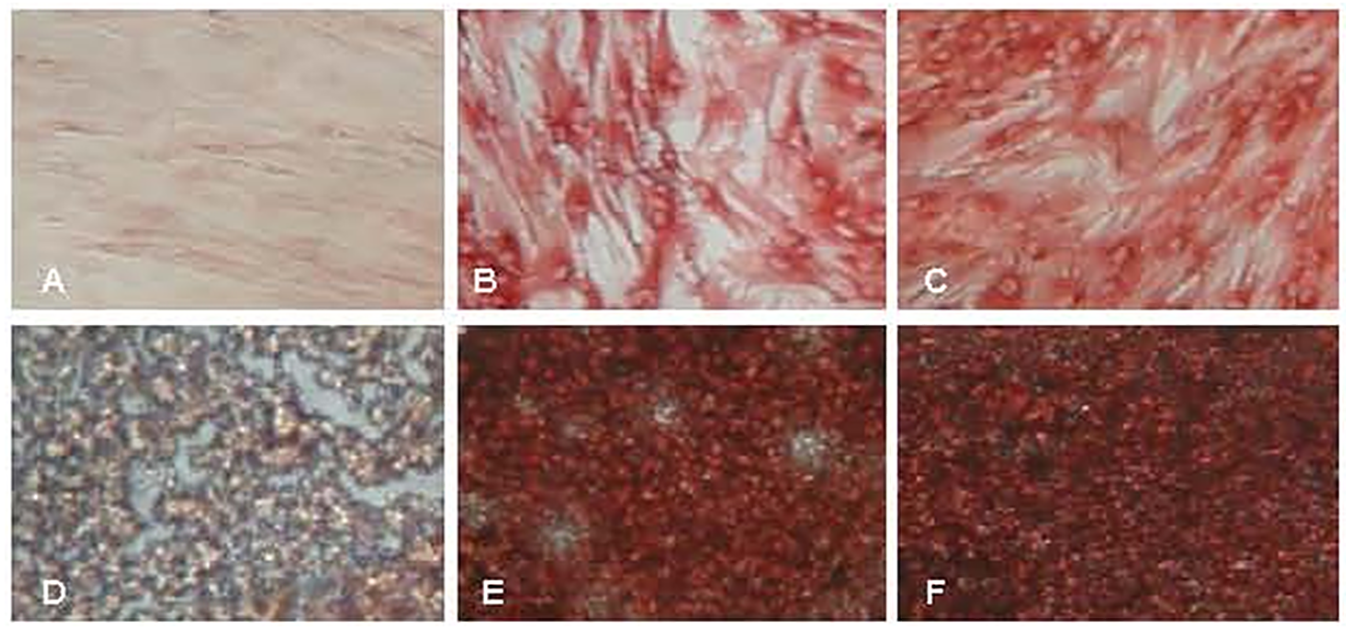
scSOX9 induced collagen type II expression. Human bone marrow derived mesenchymal stem cells (MSC) was cultured with 10 μg/ml of scGFP (A and D) or scSOX9 (B and E) or a cock tail of growth factor (GF) (C and F) in monolayer (A, B and C) or aggregate (D, E and F) for chondrogenesis. At day 14, aggregates were harvested and snap-frozen. Cryostat sections were stained with a mouse anti-human collage II monoclonal antibody. (Immunoperoxidase staining. Note the poorly formed aggregate by MSC cultured with scGFP only. Representative of 4 experiments). scSOX9: super-charged SRY-type high-mobility group box-9; scGFP: super-charged green fluorescence protein.

scSOX9 was able to induce MSC proliferation and differentiation with no requirement of additional growth factors. The number of scSOX9-treated MSC was increased two folds after 48 hours in culture compared with that scGFP only treated MSC. scSOX9 alone without addition of other growth factors was capable of inducing MSC chondrogenesis, similar to that induced by the cocktail of growth factors in the standard protocol. As early as 48 hours, scSOX9-treated MSC started to change morphology into chondrocyte like cells and this morphology maintained for 21 days in culture of the duration of experiment. Furthermore, while inducing MSC morphology change, scSOX9 also induced increased expression of SOX5 and SOX9 and collagen type II, but downregulated collagen type I and type X production (Fig 1 and Fig 2). This composition of matrix collagen types is typical characteristics of articular chondrocytes.

### scSOX9 delivery by a bilayer collagen membrane *in vitro* and *in vivo*

We next further tested the bioactivity of scSOX9 *in vivo* in a rabbit cartilage injury model. A commercial bilayer collagen membrane (Bio-Gide), was used as a carrier for scSox9 to be administered at the site of microfracture. We first tested the collagen membrane for adhesion and release of scSox9 *in vitro*. A collagen membrane was soaked in scSOX9 solution. Green fluorescence was grossly visible on the collagen membrane. By measuring scSOX9 remained in the solution, it was calculated that collagen membrane bound 40–60% of the total scSOX9. Release of scSOX9 from collagen membrane was tested by rinsing the membrane with PBS containing heparin. Over 95% of scSOX9 bound on collagen membrane was released and re-dissolved in solution.

We then tested scSOX9 function in the cartilage defect repair model in New Zealand female rabbits as described [11]. The efficiency of scSOX9 delivery into MSC *in vivo* was assessed first. A cylindrical osteochondral defect was created in patellar groove of the femur as described [11, 22, 23]. Microfracture was created and bleeding of bone marrow was allowed to fully fill the defect. A collagen membrane harboring scSOX9 was secured to cover the defect (S2 Fig). One hour later, the bone marrow clot from the defect was harvested, minced and digested with streptokinase as described [24]. On average, 30 μl of bone marrow clot was recovered from each defect (the calculated volume of each defect was 37.68 μl). The digested bone marrow cell suspension was washed with PBS containing heparin to eliminate cell membrane bound scSOX9, and stained with fluorochrome labeled monoclonal antibodies to identify MSC: CD90-APC, CD11b-PE, CD79a-PE and MHC-DR-PE (BIO-RAD). The cells were analyzed on flow cytometry for delivery of scSOX9 into MSC. As shown in S3 Fig, MSC were defined as CD90^+^/CD11b^-^/CD79a^-^/DR^-^ [32, 33] and comprised of about 0.015% of the total nucleated bone marrow cells. The frequency of MSC in our bone marrow cell preparation was consistent with previously estimated [34]. About 60% of MSC in the bone marrow clot were showing GFP positive (S3 Fig) indicating that scSOX9 entered these cells. The efficiency of scSOX9 transfection is comparable to transgene expression mediated by rAVV in MSC [35].

### Gross morphology assessment of cartilage repair

**Fig 3:**
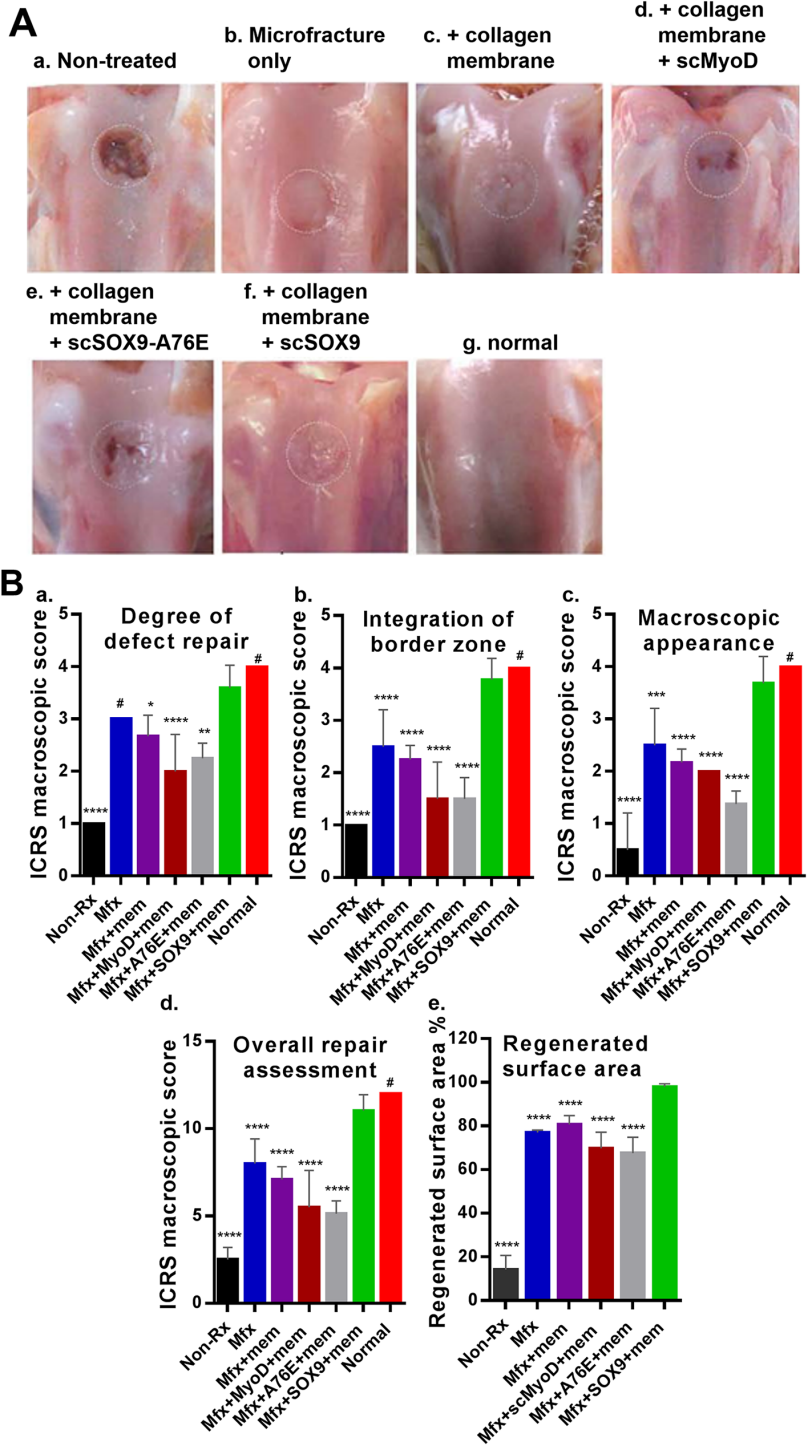
Macroscopic assessment of cartilage repair. **(A)** Photographs of rabbit knee articular osteochondral defects 8 weeks after treatments, the joints were examined grossly. The dotted circles indicate the original defect margin. Normal cartilage surface of the distal femur was used as reference (g). **(B)** Macroscopic assessment of cartilage repair was quantified by International Cartilage Repair Society (ICRS) macroscopic evaluation scale (S1 Table) [27]. Non-Rx: Non-treated; Mfx: microfracture only; Mfx + mem: microfracture and collagen membrane; Mfx+MyoD+mem: microfracture and collagen membrane containing myogenic differentiation factor (MyoD); Mfx+A76E+mem: microfracture and collagen membrane containing mutant form of super-charged SRY-type high-mobility group box-9 (scSOX9-A76E); Mfx+SOX9+mem: microfracture and collagen membrane containing scSOX9; Normal: normal cartilage. Results were presented as the mean ± SD; statistical analysis was performed to compare scores of each of other treatment groups and normal cartilage to that of Mfx+SOX9+mem group respectively; n = 4–11 in each group; *p < 0.05; ** p < 0.01; *** p < 0.001; **** p < 0.0001; # Not significant.

Gross appearance of regenerated tissue is shown in Fig 3 to demonstrate the gross morphology assessment. DNA-dependent dimerization of SOX9 is required for SOX9 to mediate chondrogenesis. A mutation of the dimerization domain, A76E results in loss of the chondrogenic property of SOX9 which causes campomelic dysplasia in human [36, 37]. In order to ensure that scSOX9 actively induce chondrognesis *in vivo*, we produced a scSOX9 with this mutation, named scSOX9-A76E as a negative control.

The defect of cartilage was either left with no treatment, treated with microfracture, microfracture with collagen membrane only, scSOX9-A76E bound collagen membrane, scMyoD bound collagen membrane, or scSOX9 bound collagen membrane. In non-treated animals, a large defect existed and the original defect area was uncovered (Fig 3A, a). In those treated with microfracture only, the defect was largely filled with white soft tissue and the boundary between normal cartilage and regenerated tissue was distinct (Fig 3A, b). Defect in microfracture with collagen membrane only treated had better coverage (Fig 3A, c). In microfracture plus scMyoD treated, vacant position still existed and a part of the defect was covered by uneven, a thin layer of fibrous tissue. Fissures existed on the surface of newly formed tissue in the defect (Fig 3A, d). Similarly, in microfracture plus scSOX9-A76E showed poor coverage of the defect (Fig 3A, e). In contrast, in microfracture plus scSOX9 treated, the entire original defect was filled with glossy semitransparent repaired tissue that appeared to be smooth and well-integrated with the surrounding tissues. The color and thickness of regenerated tissue more resembled the adjacent normal cartilage (Fig 3A, f). The degree of cartilage repair was assessed by ICRS macroscopic assessment scale for cartilage repair. This composite score system consists of assessment of degree of defect repair, integration to border zone and macroscopic appearance. The degree of cartilage repair was quantified by ICRS score system and was compared between treatment groups as shown in Fig 3B. The higher total ICRS scores were seen in microfracture plus scSOX9 treated group than any other groups (Fig 3B) indicating that microfracture combined with scSOX9 significantly improved cartilage regeneration. The percentage of regenerated tissue in the defect area was shown in Fig 3B, e.

### Histological assessment of cartilage repair

**Fig 4:**
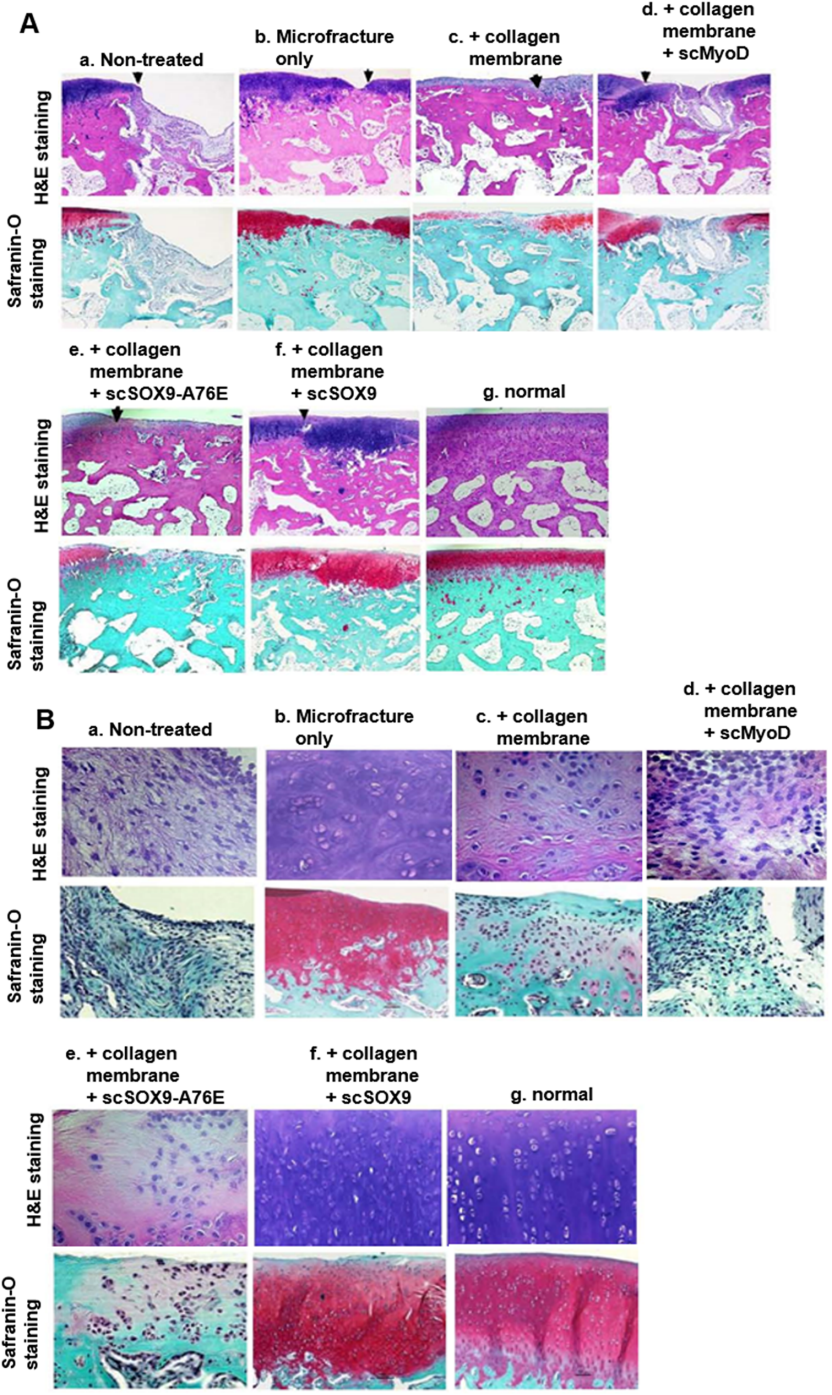
Histological analysis of reparative cartilage. The distal femurs were fixed in 10% formalin, decalcified, embedded in paraffin, and cut into 5 μm sections. Sections from each sample were then stained with hematoxylin and eosin (H&E) for morphological evaluation (**A**, low magnification (x 40) and **B**, high magnification (H&E stating x 200; Safranin O staining x 100). Safranin O and fast green staining was used to assess matrix proteoglycan distribution (**A** and **B**). Note that non-treated cartilage injury did not grow cartilage-like tissue to the surface of cartilage (**B, a**) (n = 4–11 in each group).

After gross examination, all specimens were processed for paraffin embedding. Sections were stained with hematoxylin and eosin and Safranin O and fast green (Fig 4A and 4B) using standard protocols. Histological analysis showed that the quality of the regenerated tissues differed depending on the treatment. As shown in Fig 4A, a, the joint surface of the defects in non-treated group was still concave. A large area defect existed and was covered with little tissue. In microfracture treated only (Fig 4A, b), the defect was filled with a larger amount of cartilage-like tissue with cells arranged disorderly and the gap between the normal cartilage and regenerated tissue was apparent. The surface was irregular and concave areas could be observed. Safranin O staining was less positive. In collagen membrane treated, tissue appeared to be fibrous like tissue with little or no Safranin O stating (Fig 4A, c; Fig 4B, c). In microfracture plus scMyoD treated, the defect was filled with a mixture of fibrous tissue and cartilage-like tissue as shown by hematoxylin and eosin and safranin O staining. The surface of the defects was still concave, irregular or empty in the middle. Safranin O was partially and slightly positive or negative (Fig 4A, d; Fig 4B, d). In scSOX9-A76E treated group, some of the samples had partial coverage (Fig 4A, e), histologically, these appeared cartilage like tissue and mixed with fibrous tissue (Fig 4A, e; Fig 4B, e). In non-treated, microfracture only and microfracture plus scMyoD or scSXO9-A76E treated, the boundary between normal cartilage and regenerated tissue was distinct. Whereas, in microfracture plus scSOX9 treated (Fig 4A, f; Fig 4B, f), the defects were mostly filled with hyaline-like cartilage with a regular surface that was well-integrated with the native cartilage. The regenerated tissue thickness was similar to the surrounding normal tissue. Hyaline-like cartilage regenerated tissue filled the entire defect. However, the tide mark is not continuous compared to that in normal cartilage suggesting that repairing of the cartilage and subchondral bone is incomplete or tissue remodeling is still ongoing. The junction gap was not distinct. An abundance of cartilage matrix could be identified by Safranin O staining (Fig 4A, f; Fig 4B, f). Subchondral bone bridged over the defect in scSOX9 group.

The histological appearance of the repaired tissue in the scSOX9 group was significantly improved compared to that of the other groups. For further identification of regenerated cartilage in different groups, high magnification observation and normal femur section stained with hematoxylin and eosin and Safranin O were used for comparison (Fig 4B). The repaired tissues in microfracture group were predominantly fibrocartilage (Fig 4B, b), as they were slightly stained with Safranin O (Fig 4B, b). In contrast, the scSOX9 group contained defects filled with hyaline-like cartilage that was stained with Safranin O and chondrocytes and cartilage lacuna existed (Fig 4B, f). The chondrocyte cell morphology, structural integrity, and thickness of the positively stained tissue in the scSOX9 group more closely resembled normal articular cartilage (Fig 4B, f, Fig 4B, g). Nonetheless, subtle difference between normal cartilage and scSOX9 induced reparative tissue existed. The cellular density in scSOX9 induced reparative tissue was higher than that in normal cartilage.

**Fig 5:**
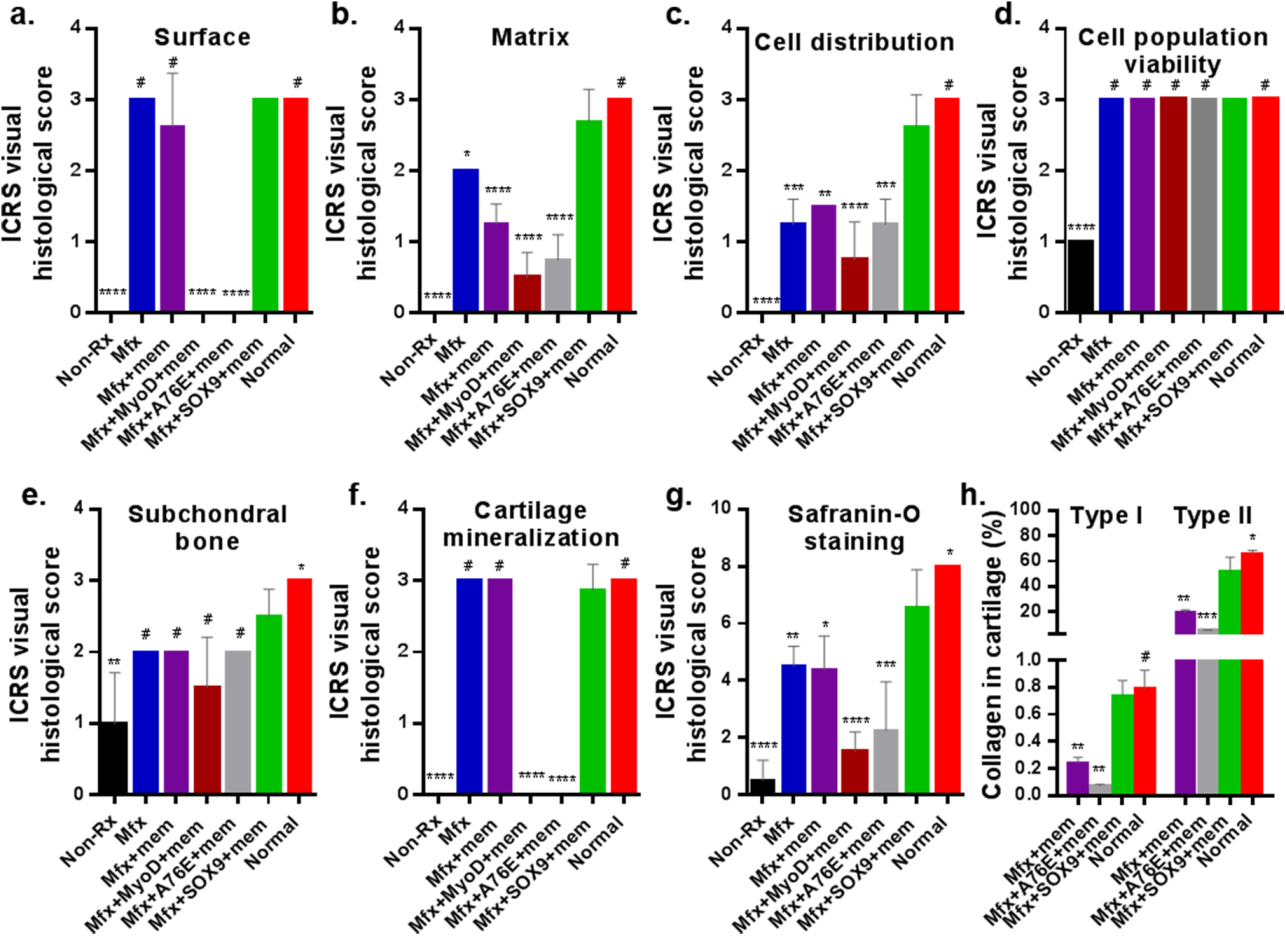
International Cartilage Repair Society (ICRS) scores and the percentage of regenerated tissue. H&E staining sections were scored according to ICRS assessment system (S2 Table) and updated recommendations [28, 29]. Each of the categories of assessment (**a, b, c, d, e and f**) was reported separately as recommended by ICRS [28]. **g:** Quantification of Safranin O staining was assessed as described previously [30]. **h:** Collagen type I and type II of the reparative cartilage tissue were quantified by ELISA and expressed as percentage of dry weight of cartilage. *p < 0.05; **p < 0.01; *** p < 0.001; ****p < 0.0001; # Not significant. (Results were presented as the mean ± SD; n = 4–11 in each group). Non-Rx: Non-treated; Mfx: microfracture only; Mfx + mem: microfracture and collagen membrane; Mfx + MyoD+mem: microfracture and collagen membrane containing myogenic differentiation factor (MyoD); Mfx+A76E+mem: microfracture and collagen membrane containing mutant form of super-charged SRY-type high-mobility group box-9 (scSOX9-A76E); Mfx+SOX9+mem: microfracture and collagen membrane containing scSOX9; Normal: normal cartilage. Results were presented as the mean ± SD; statistical analysis was performed to compare scores of each of other treatment groups and normal cartilage to that of Mfx+SOX9+mem group respectively; n = 4–11 in each group; *p < 0.05; ** p < 0.01; *** p < 0.001; **** p < 0.0001; # Not significant.

The overall tissue regeneration was quantified according to the ICRS Visual Histological Assessment Scale as described [28, 29]. It is required that scores in each of the category is reported separately and should not be summed [28]. Scores of the ICRS visual histological scale of reparative cartilage were shown in Fig 5. The microfracture plus scSOX9 treated group led to highest ICRS scores than any other group in the key components (Fig 5B, 5C and 5G). Despite that microfracture and microfracture plus collagen membrane only achieved high scores in Category “Surface”, which were comparable to that treated with microfracture plus scSOX9, the reparative tissue in scSOX9 treated group had substantial higher scores in matrix and cell distribution (Fig 5B and 5C). Categories “Matrix” and “Cell distribution” assess whether the reparative tissue was resembling hyaline cartilage (S2 Table). “Cell population viability” assesses reparative tissue cell viability. There was no difference found between treatment groups (Fig 5D). “Subchondral bone” assesses subchondral bone remodeling. There was no difference noted between treatment groups, but statistical difference was found between normal cartilage and the microfracture plus scSOX9 treated group (Fig 5E). This reflected effect of microfracture which induces subchondral bone remodeling process during cartilage repair. Cartilage mineralization within cartilage is a pathological phenomenon. Cartilage mineralization was only noted in treatment groups of MyoD and scSOX9-A76E (Fig 5F). Cartilage matrix contents of reparative tissue were assessed by Safranin O staining and quantification of collagen types. The intensity of Safranin O staining directly reflects the amount of proteoglycan. Microfracture plus scSOX9 treated achieved highest score compared with that in other treatment groups, but the score was statistically significantly lower than that of normal cartilage (Fig 5G). This suggests that reparative cartilage by microfracture plus scSOX9 treatment contained a lower amount of proteoglycan than normal cartilage although the difference was small. We next quantified content of collagen type I and type II in reparative tissue. As shown in Fig 5H, the reparative tissue treated with microfracture combined with scSOX9 had highest content of collagen type II and the ratio of type II and type I was similar to that in normal cartilage. All these results indicated that microfracture combined with scSOX9 induced a type of reparative tissue most closely resembled hyaline cartilage.

Using MSC has been an attractive approach to articular cartilage repair [5] which is commonly conducted via gene transfer. Studies of gene transfer are typically of cell based and in combination with tissue engineering. MSC are harvested from bone marrow, adipose tissue or synovium of the joint and expanded and transfected *in vitro* with one of the chrondrogenic growth factors. Then the modified MSC are transferred to the site of cartilage defect. Several preclinical studies with animal models of cartilage injury have reported successful growth of hyaline cartilage-like reparative tissue. But drawbacks have been associated with gene therapy for cartilage repair. Gene therapy procedures are rather complex with *in vitro* culture and transfection of MSC with vectors carrying candidate genes. Currently most effective gene transfer is achieved with viral vectors, such as adenovirus, retrovirus, lentivirus and recombinant adeno-associated virus (rAAV) [35]. Adenovirus is highly efficient for gene transfer but is toxic and immunogenic. Retrovirus and lentivirus are also highly efficient and can provide with long term transgene expression, but have risk of insertional mutagenesis. Therefore, adenovirus, retrovirus and lentivirus are not candidates for moving forward to use in humans. rAAV vectors offer very high efficiency and long-term transgene expression with low immunogenicity. Moreover, rAAV vectors are capable of modifying the cells in situ in the extracellular matrix environment. Recently, Cucchiarini and Madry reported use of rAAV vector mediated direct gene transfer for over expression of human insulin-like growth factor-1 (IGF-1) to improve early repair of articular cartilage defects *in vivo* [38]. Thus, rAAV has become a promising vector for clinical applications. But rAAV vectors are difficult to produce with limitation of gene size to be transferred and potential serotype-restricted cell specificity [38]. On the other hand, non-viral gene transfer combined with tissue engineering has been investigated for effects of overexpression of potential therapeutic genes in MSC. The major issues with non-viral gene transfer have been low efficiency and complicated *in vitro* procedure [39, 40].

Here we report a novel strategy for articular cartilage repair using cutting-edge molecular technology in combination with a procedure commonly performed in clinical practice. Bone marrow derived MSC have the potential to form a variety of mesenchymal tissues including bone, tendon, ligament, muscle and fat depending on the presence of cytokines or growth factors [41]. *In vitro* studies have demonstrated that many growth factors are capable of promoting chondrogenic differentiation of MSC. These include IGF-1, fibroblast growth factor-2, bone morphogenetic proteins, transforming growth factor-ß (reviewed in [5]). Interestingly, all these growth factors induce chondrogenesis of MSC via stimulation of SOX9 expression. SOX9 is transported actively into the nucleus through the two distinct NLS and interact with a number of genes that are involved in chondrogenesis. The cell penetrating protein, scSOX9 efficiently entered MSC and induced chondrogenesis. SOX9 nudges MSC towards chondrocytes. Maintenance of chondrocytes requires the continuous expression of endogenous SOX9 and other transcription factors such as SOX5 and/or SOX6 which co-operate with SOX9 in chondrogenesis [13]. Our results are in consistence with this notion. Thus, scSOX9 could induce endogenous SOX9 and SOX5 gene expression in MSC *in vitro* culture, but did not significantly induce SOX6 expression at 24 and 48 hours. In a recent study, Liu and Lefebvre [42] analyzed cooperation between SOX9 and SOX5/SOX6 at genome-wide level during chondrogenesis and revealed great overlap between SOX9 and SOX6 in binding to super-enhancers associated with cartilage specific genes. SOX5 and SOX6 have largely redundant functions in chondrogenesis. SOX9, with either SOX5 or SOX6 is sufficient to induce chondrogenesis. In our study, scSOX9 preferentially induces SOX5 instead of SOX6. This interesting observation deserves further study, in particular in *in vivo* studies.

The ultimate hyaline-like cartilage induction by scSOX9 *in vivo* suggests that the same mechanism was in operation during cartilage repair. However, this remains to be confirmed. Chondrogenesis from MSC *in vitro* indicate that SOX9 is the master transcription factor and this was confirmed by our study with scSOX9. It remains unknown whether scSOX9 is able to turn on chondrogenesis of other cell types *in vivo*. Indeed, scSOX9 was able to enter other cell types in the bone marrow other than MSC, but may not be able to induce chondrogenesis since scSOX9 failed to induce chondrogenesis from fibroblasts. Nevertheless, it is likely that scSOX9 administered at the site of cartilage injury may be able to enter adjacent chondrocytes and chondrocyte progenitors at the superficial layer of cartilage. These progenitors have potential to generate chondrocytes *de novo* [43]. It is possible that scSOX9 also induce these chondrocyte progenitors to differentiate into chondrocytes and participate in cartilage repair. Our knowledge about kinetics of SOX9 expression and its regulation of other genes in cartilage repair is mostly extrapolated from cartilage development and *in vitro* cell culture studies [6–8, 14, 42]. It is largely unknown *in vivo* at what time point during MSC differentiation into chondrocytes SOX9 gene expression will be turned down. Our *in vitro* data demonstrated that scSOX9 maintained in the MSC for up to 7 days (Fig F in S1 Fig) However, it is not clear whether this will truly reflect the status of scSOX9 *in vivo*. Our on-going effort is to address this issue *in vivo* in our rabbit model of cartilage injury and repair by assessing persistence of exogenous scSOX9 and endogenous SOX9 expression by microarray analysis of gene expression by MSC and regenerated chondrocytes at different time points after administration of scSOX9. Our *in vivo* model would allow further analysis of the kinetics of SOX9 and SOX5/SOX6 expression induced by scSOX9 during cartilage repair and elucidation of interactions between scSOX9, and endogenous SOX9, SOX5 and SOX6.

## Conclusions

Regeneration of hyaline cartilage has been an attractive approach to cartilage repair. However, current cartilage transplants and MSC based methods have significant drawbacks. Our approach took advantage of non-vial delivery of chondrogenesis master transcription factor into bone marrow-derived MSC which are recruited to the site by microfracture. Microfracture is an easy, simple, single stage procedure with low morbidity and low cost. By local application of cell penetrating SOX9, we were able to significantly improve the quality of microfracture induced cartilage repair. By morphological assessment, it is likely that scSOX9 had enhanced the quality of microfracture by promoting MSC contained in bone marrow clot into chondrocytes *in situ*. Quantitative measurement of collagen type I and II contents indicate the reparative cartilage tissue was consistent with hyaline cartilage. From the morphology analysis, the reparative cartilage induced by scSOX9 is not completely matching the normal articular cartilage. The non-continuous tide mark and relatively higher chondrocyte density suggest continuous tissue remodeling. Nonetheless, our approach has substantially improved the short-term efficacy of microfracture in generating genuine hyaline-like cartilage *in situ* although long-term observation is required to confirm the cartilage generated in this method is durable.

## Supporting information

S1 Table ICRS macroscopic evaluation of cartilage repair. (PDF)

S2 Table ICRS Visual Histological Assessment Scale. (PDF)

S1 Fig Penetration of scSOX9 protein into HFF and MSC. (PDF)

S2 Fig **Schematic diagram** showing a model of cartilage defect (A), microfracture (B) and application of scSOX9 in a collagen membrane (C) for cartilage repair.
(PDF)

S3 Fig Efficiency of scSOX9 delivery into MSC *in vivo*. (PDF)
