# Supplementary material for: Regeneration of hyaline-like cartilage in situ with SOX9 stimulation of bone marrow-derived mesenchymal stem cells
Source: PLoS One. 2017 Jun 30;12(6):e0180138. doi: 10.1371/journal.pone.0180138 (PMC5493350; doi:10.1371/journal.pone.0180138)
Supplement: S1 Table — (PDF) [file pone.0180138.s001.pdf]

S1 Table. ICRS macroscopic evaluation of cartilage repair

Cartilage repair assessment ICRS	Points
Degree of defect repair	
In level with surrounding cartilage	4
75% repair of defect depth	3
50% repair of defect depth	2
25% repair of defect depth	1
0% repair of defect depth	0
Integration to border zone	
Complete integration with surrounding cartilage	4
Demarcating border < 1 mm	3
3/4th of graft integrated, 1/4th with a notable border > 1 mm width	2
1/2 of graft integrated with surrounding cartilage, 1/2 with a notable border > 1 mm	1
From no contact to 1/4th of graft integrated with surrounding cartilage	0
Macroscopic appearance	
Intact smooth surface	4
Fibrillated surface	3
Small, scattered fissures or cracks	2
Several, small or few but large fissures	1
Total degeneration of grafted area	0
Overall repair assessment	
Grade I: normal	12
Grade II: nearly normal	11–8
Grade III: abnormal	7–4
Grade IV: severely abnormal	3–1
