# Supplementary material for: Regeneration of hyaline-like cartilage in situ with SOX9 stimulation of bone marrow-derived mesenchymal stem cells
Source: PLoS One. 2017 Jun 30;12(6):e0180138. doi: 10.1371/journal.pone.0180138 (PMC5493350; doi:10.1371/journal.pone.0180138)
Supplement: S2 Table — (PDF) [file pone.0180138.s002.pdf]

S2 Table. ICRS Visual Histological Assessment Scale

Feature	Points
I. Surface	
Smooth/continuous	3
Discontinuities/irregularities	0
II. Matrix	
Hyaline	3
Mixture: hyaline/fibrocartilage	2
Fibrocartilage	1
Fibrous tissue	0
III. Cell distribution	
Columnar	3
Mixed/columnar-clusters	2
Clusters	1
Individual cells/disorganized	0
IV. Cell population viability	
Predominantly viable	3
Partially viable	1
<10% viable	0
V. Subchondral Bone	
Normal	3
Increased remodeling	2
Bone necrosis/granulation tissue	1
Detached/fracture/callus at base	0
VI. Cartilage mineralization (calcified cartilage)	
Normal	3
Abnormal/inappropriate location	0
Safranin O stain	
Normal	4
Slight reduction	3
Moderate reduction	2
Severe reduction	1
No staining	0
Percent safranin O in defect	
75–100%	4
50–75%	3
25–50%	2
0–25%	1
No safranin O staining	0
