# Supplementary material for: Regeneration of hyaline-like cartilage in situ with SOX9 stimulation of bone marrow-derived mesenchymal stem cells
Source: PLoS One. 2017 Jun 30;12(6):e0180138. doi: 10.1371/journal.pone.0180138 (PMC5493350; doi:10.1371/journal.pone.0180138)
Supplement: S1 Fig — (PDF) [file pone.0180138.s003.pdf]

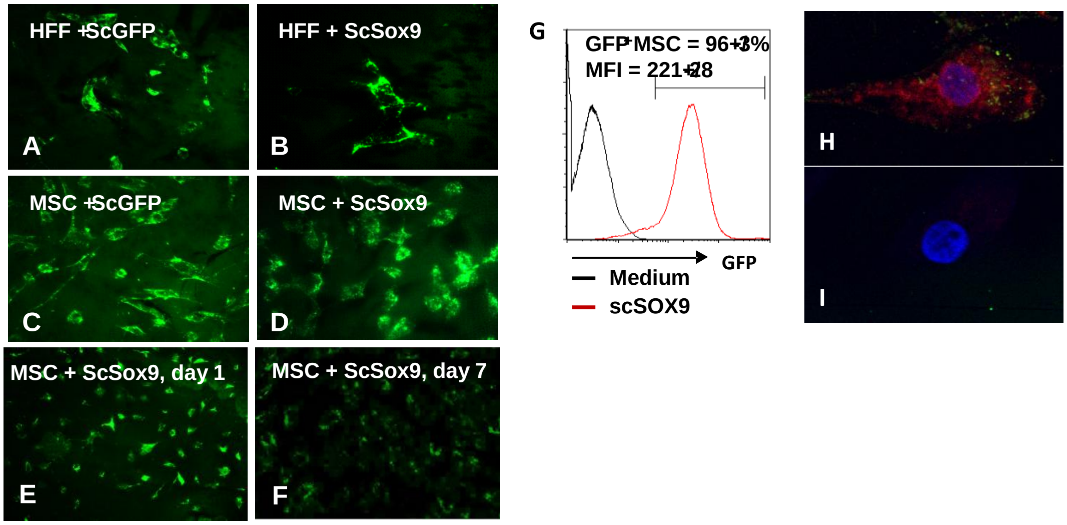

S1 Fig. Penetration of scSOX9 protein into HFF and MSC. Human skin fibroblast cell line, HFF (A and B) or human bone marrow MSC (C and D) were incubated with 10 µg/ml of supercharged green fluorescence protein (scGFP) or SOX9-scGFP fusion protein (scSOX9) in DMEM at 37°C for 1 hour. Cells were washed with PBS containing heparin to remove cell membrane bound scSOX9 and viewed under fluorescent microscope. A and C: scGFP; B and D: scSOX9. E and F: MSC cultured with scSOX9 as in D for 1 day and 7 days. G. MSC were treated scSOX9 as in D or with medium alone. Cells were washed and detached with trypsin and analyzed in flow cytometry. MFI, mean fluorescence intensity. H and I: MSC were grown in monolayer with scSOX9 (H) or medium alone (I). Cells were washed with PBS containing heparin and permeabilized, followed by incubation with anti-His-biotin and streptavidin-Cy3 (red) and viewed under a confocal microscope. The nucleus was stained blue with DAPI. Representative of 3 experiments.
