# Supplementary material for: Regeneration of hyaline-like cartilage in situ with SOX9 stimulation of bone marrow-derived mesenchymal stem cells
Source: PLoS One. 2017 Jun 30;12(6):e0180138. doi: 10.1371/journal.pone.0180138 (PMC5493350; doi:10.1371/journal.pone.0180138)
Supplement: S2 Fig — Schematic diagram showing a model of cartilage defect (A), microfracture (B) and application of scSOX9 in a collagen membrane (C) for cartilage repair. (PDF) [file pone.0180138.s004.pdf]

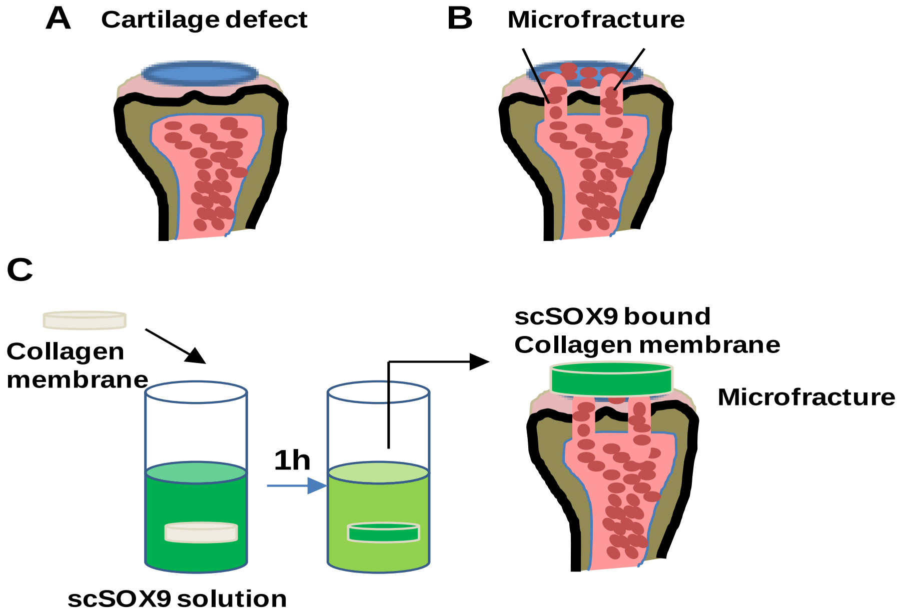

S2 Fig. Schematic diagram showing a model of cartilage defect (A), microfracture (B) and application of scSOX9 in a collagen membrane (C) for cartilage repair (this diagram was prepared based on Fig. 1 of reference #25 with modifications).
