# Supplementary material for: Regeneration of hyaline-like cartilage in situ with SOX9 stimulation of bone marrow-derived mesenchymal stem cells
Source: PLoS One. 2017 Jun 30;12(6):e0180138. doi: 10.1371/journal.pone.0180138 (PMC5493350; doi:10.1371/journal.pone.0180138)
Supplement: S3 Fig — (PDF) [file pone.0180138.s005.pdf]

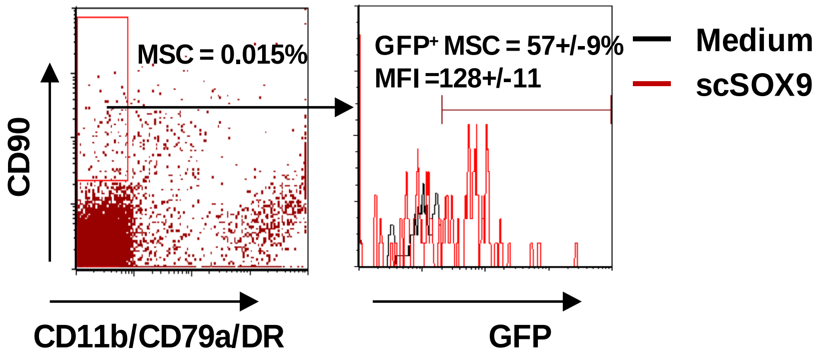

S3 Fig. Efficiency of scSOX9 delivery into MSC *in vivo*. Osteochondral defect was created and microfracture was performed. One hour after administration of scSOX9, the bone marrow clot was harvested and digested. The cell suspension was washed with PBS containing 20 units of heparin to wash off possible cell membrane bound scSOX9, then stained with a cocktail of PE-labeled monoclonal antibodies to CD11b, CD79a, MHC-DR and APC labeled anti-CD90, and analyzed in flow cytometry. MSC was defined as $CD90^+/CD11b^-/CD79a^-/DR^-$. GFP positive cells indicated that scSOX9 entered MSC (Representative of 3 experiments).
